# Ectopic expression of the sesame MYB transcription factor *SiMYB305* promotes root growth and modulates ABA-mediated tolerance to drought and salt stresses in *Arabidopsis*

**DOI:** 10.1093/aobpla/plz081

**Published:** 2019-12-24

**Authors:** Komivi Dossa, Marie A Mmadi, Rong Zhou, Aili Liu, Yuanxiao Yang, Diaga Diouf, Jun You, Xiurong Zhang

**Affiliations:** 1 Oil Crops Research Institute of the Chinese Academy of Agricultural Sciences, Key Laboratory of Biology and Genetic Improvement of Oil Crops, Ministry of Agriculture, No.2 Xudong 2nd Road, Wuhan, China; 2 Laboratoire Campus de Biotechnologies Végétales, Département de Biologie Végétale, Faculté des Sciences et Techniques, Université Cheikh Anta Diop, BP 5005 Dakar-Fann, Dakar, Sénégal

**Keywords:** Abiotic stress, abscisic acid, MYB transcription factors, root growth, *Sesamum indicum*, *SiMYB75*

## Abstract

An increasing number of candidate genes related to abiotic stress tolerance are being discovered and proposed to improve the existing cultivars of the high oil-bearing crop sesame (*Sesamum indicum* L.). However, the *in planta* functional validation of these genes is remarkably lacking. In this study, we cloned a novel sesame R2-R3 MYB gene *SiMYB75* which is strongly induced by drought, sodium chloride (NaCl), abscisic acid (ABA) and mannitol. *SiMYB75* is expressed in various sesame tissues, especially in root and its protein is predicted to be located in the nucleus. Ectopic over-expression of *SiMYB75* in *Arabidopsis* notably promoted root growth and improved plant tolerance to drought, NaCl and mannitol treatments. Furthermore, *SiMYB75* over-expressing lines accumulated higher content of ABA than wild-type plants under stresses and also increased sensitivity to ABA. Physiological analyses revealed that *SiMYB75* confers abiotic stress tolerance by promoting stomatal closure to reduce water loss; inducing a strong reactive oxygen species scavenging activity to alleviate cell damage and apoptosis; and also, up-regulating the expression levels of various stress-marker genes in the ABA-dependent pathways. Our data suggested that *SiMYB75* positively modulates drought, salt and osmotic stresses responses through ABA-mediated pathways. Thus, *SiMYB75* could be a promising candidate gene for the improvement of abiotic stress tolerance in crop species including sesame.

## Teaser

In this study, the gene SiMYB75 was cloned from sesame (*Sesamum indicum* L.) and was found to be strongly induced by drought, sodium chloride (NaCl), abscisic acid (ABA) and mannitol. SiMYB75 displayed a root preferential expression and the ectopic over-expression in *Arabidopsis thaliana* demonstrated that it promotes root growth and is involved in drought and salinity tolerance by modulating the expression of ABA-related genes and strongly reducing reactive oxygen species production in cells. SiMYB75 could be a promising candidate gene for the improvement of abiotic stress tolerance in crop species including sesame..

## Introduction

Unfavourable environmental conditions, including extreme temperatures, flooding, drought and high salinity limit crop growth and productivity resulting in considerable yield loss worldwide. Hence, improving crop tolerance to environmental stresses is of a paramount significance for global food security. Biotechnological manipulation of genes with high potential to impart cellular processes of stress tolerance is among the strategies for improving crop tolerance (Seok *et al.* 2017).

To protect themselves from abiotic stresses, plants translate environmental inputs into internal signals through hormones, second messengers which activate transcription factors (Wilkinsonet and Davies 2002; Shinozaki and Yamaguchi-Shinozaki 2007). In general, plants respond to abiotic stresses through abscisic acid (ABA)-dependent pathway and ABA-independent pathway. ABA is a broad-spectrum phytohormone that coordinates various stress signal transduction pathways during abiotic stress responses in plants (Agarwal and Jha 2010). Transcription factors (TFs) are the key mediators of these stress signal transduction pathways by regulating the expression of downstream target genes (Shinozaki *et al.* 2003). Several classes of TFs such as *Apetala* 2/ethylene-responsive element binding factor (AP2/ERF), NAM/ATAF1/CUC2 (NAC), WRKY, basic leucine zipper (bZIP), basic helix-loop-helix (bHLH), C2H2 zinc fingers (ZFs) and Myeloblastosis (MYB) were identified as involved in abiotic stress responsive pathways in plants (Abe *et al.* 1997; Nakashima *et al.* 2007; Kodaira *et al.* 2011; Jisha *et al.* 2015; Tu *et al.* 2016;Zhao *et al.* 2018).

MYBs are a vital gene family with a large number of members which modulate various biological processes in plants such as shoot growth, root formation, organ development, metabolism, hormone signal transduction and response to biotic and abiotic stresses (Jin and Martin 1999; Dubos *et al.* 2010; Ambawat *et al.* 2013; Roy 2016). They have attracted tremendous investigations in various plant species mainly regarding their involvement in plant abiotic stresses tolerance. The MYB domain is composed of one to four imperfect tandem repeats (R) and each repeat adopted a helix-turn-helix (HTH) conformation with 51–53 amino acid residues (Stracke *et al.* 2001). MYB proteins are classified into four major subfamilies based on the number of imperfect adjacent repeats in the MYB domain: R1-MYB or MYB-related (1R), R2R3-type MYB (2R), R1R2R3-type MYB (3R) and 4R-type MYB or atypical MYB (4R). Among them, the R2R3-MYB subfamily is the most abundant in plants (Stracke *et al.* 2001). In the model plant species *Arabidopsis thaliana* which contains 198 MYB genes, the functional characterization of the *AtMYB96* gene showed that its over-expression increases cuticular wax biosynthesis under drought conditions as a tolerance mechanism (Lee *et al.* 2014). Moreover, several other genes including *AtMYB44*, *AtMYB73*, *AtMYB20*, *AtMYB2* and *AtMYB15* were reported to confer drought and salt tolerance through ABA-mediated pathways (Jung *et al.* 2008; Ding *et al.* 2009; Cui *et al.* 2013; Kim *et al.* 2013). Ectopic over-expression of the rice gene *OsMYB3R-2* confers resistance to multiple abiotic stresses in *Arabidopsis* (Dai *et al.* 2007). Similarly, Yin *et al.* (2017) characterized the rice gene *OsMYBR1* and found that transgenic *Arabidopsis* plants up-regulate many stress-related genes and strongly accumulate osmoprotectants, leading to improved drought tolerance. Recent studies of Butt *et al.* (2017) and Li *et al*. (2017b) revealed that two novel genes *PbrMYB21* from *Pyrus betulaefolia* and *GaMYB85* from *Gossypium arboreum* control the polyamine levels and stomata density as key mechanisms for drought tolerance.

Sesame (*Sesamum indicum* L.) is a traditional oilseed crop highly valued for its health-promoting oil (Anilakumar *et al.* 2010). With the growing interest in this crop, the global production and the area down to sesame are rapidly increasing. However, sesame is yet to become a major crop in the world because of the weak productivity, the lack of improved varieties with tolerance to biotic and abiotic stresses (Dossa *et al.* 2017a). Sesame is mainly grown in arid and semi-arid areas and is often challenged by severe drought (Langham 2007). Although sesame is rated as a relatively drought tolerant crop, the plant growth and yield are critically affected under prolonged stress (Sun *et al.* 2010;Dossa *et al.* 2017b). Similarly, adverse effects of salt stress, especially NaCl, on sesame seed germination, seedling growth and yield were reported (Koca *et al.* 2007; Yahya 2010; Bazrafshan and Ehsanzadeh 2014; Bekele *et al.* 2017; Li *et al.* 2018). Gene mining for improvement for abiotic stress tolerance in sesame, particularly, towards drought and salt stress tolerance represents the trending issue in the current sesame research (Dossa *et al.* 2017a). An increasing number of abiotic stress tolerance candidate genes are being discovered in sesame (Dossa *et al*. 2016a, b, c; Wang *et al.* 2016; Dossa *et al.* 2017c; Li *et al*. 2017b; Mmadi *et al.* 2017;Li *et al.* 2018; Wang *et al*. 2018b; You *et al.* 2018; Zhang *et al.* 2018; Dossa *et al.* 2019a), but their functional validation is notably lacking (Chowdhury *et al.* 2017; Dossa *et al.* 2019b). Sesame resilience to the genetic manipulation is still significantly enough to justify the use of a heterologous system such as *Arabidopsis thaliana*.

In a previous report, Mmadi *et al.* (2017) identified at the genome-wide level, 287 MYB encoding genes, which were predicted to be involved in various biological pathways in sesame. They identified 28 MYB genes strongly affected by drought and waterlogging stresses. In this study, we cloned one of these genes (*SiMYB75*) and characterized its function in mediating drought and salt stress tolerance. *SiMYB75* was induced by drought, salt, ABA and osmotic stresses in sesame. Its ectopic over-expression in *Arabidopsis* promoted root growth, increased the endogenous ABA level, induced ABA sensitivity phenotype and enhanced tolerance to drought and salt stresses. Our results indicate that *SiMYB75* functions through the ABA-dependent pathways and is a potential gene for the genetic improvement of sesame abiotic stress tolerance.

## Methods

### Bioinformatics analysis of *SiMYB75*

The whole gene, promoter, coding and protein sequences of *SiMYB75* (*SIN_1015311*) were obtained from Sinbase (http://ocri-genomics.org/Sinbase/; (Wang *et al.* 2014)). The Pfam 26.0 (http://pfam.xfam.org/) database was exploited to identify the putative MYB conserved domains in the SiMYB75 protein. The theoretical Molecular weight and Isoelectric point of the SiMYB75 protein were predicted using the Compute pI/Mw tool (www.web.expasy.org/compute_pi). The promoter region (1 Kb) of *SiMYB75* and *AtMYB71* were analysed using the PLACE online tool (Higo *et al.* 1998). Next, using BLASTp search with an e-value ≤ 1E^-10^ and amino acid sequence >100 residues, we identified the homologues of SiMYB75 protein in closely related species, viz. potato, grape, oleaster, jatropha, rice and in the model plant species *Arabidopsis*. The amino acid sequences were aligned with Clustal W in the MEGA 7.0 software (Kumar *et al.* 2016) and the result was used to construct a Neighbor Joining phylogenetic tree with 1000 bootstrap replicates.

### Sesame materials and stress treatments

The sesame modern cultivar Zhongzhi No. 13 was obtained from the Sesame Germplasm Resource Preserving Center, of the Oil Crops Research Institute-Chinese Academy of Agricultural Sciences (Wuhan, China). The seeds were sterilized with 3 % sodium hypochlorite for 7 min and washed three times using sterile water. For the drought experiment, the seeds were sown in pots containing loam soil mixed with 10 % vermiculite and plants were regularly watered. After 2 weeks, the seedlings were submitted to a water stress for 7 days. Concerning the salt and osmotic stress treatments, seedlings were hydroponically grown in a box containing half-strength Hoagland solution for 2 weeks. Then, seedlings were transferred into a new nutrient solution containing 200 mM sodium chloride (NaCl) for 48 h (salt stress treatment) and a nutrient solution containing 2 % PEG6000 for 5 days (osmotic stress treatment). For abscisic acid (ABA) treatment, 1 week-old seedlings initially grown in a box containing half-strength Hoagland solution were transferred into a nutrient solution containing 100 µM ABA for 12 h. The whole experiment was conducted in a greenhouse with the temperature and relative humidity kept at 35 °C and 60 %, respectively, and under long-day conditions (16 h day/8 h night). Root samples of stressed and control plants were collected simultaneously at the end of each stress treatment.

### qRT–PCR expression profiling of *SiMYB75* under various abiotic stress treatments in sesame

RNAs from root of stressed and control sesame plants were extracted using the EASYspin Plus kit (Aidlab Biotechnologies, China) according to the manufacturer’s instructions. The RNA was treated with DNaseI and reverse transcribed with oligo (dT23) primer using the FastQuant RT kit (Tiangen Biotech, China). The *SiMYB75* specific primer pairs (**Table S1**) were designed using the Primer5.0 software (Lalitha 2000). The quantitative reverse transcriptase polymerase chain reaction analysis of *SiMYB75* was performed as described by Dossa *et al.* (2018) using the ChamQ SYBR qPCR Master Mix (Vazyme Biotec, China) on a Light Cycler 480 II (Roche, Switzerland). The relative expression level of *SiMYB75* was normalized to the expression level of the sesame *Actin 7* gene (*SIN_1006268*) based on the 2^-∆∆Ct^ method (Livak and Schmittgen 2001). This analysis was carried out in three independent biological replicates and three technical replicates of each biological replicate.

### Tissue expression analysis of *SiMYB75* in sesame

The expression pattern of *SiMYB75* in various tissues was analysed using the transcriptome sequencing data generated in our group from root, stem tip, leaf and seed of cv. Zhongzhi No. 13 under normal growth conditions (Wei *et al.* 2011).

### Vector construction and *Arabidopsis* genetic transformation

To functionally characterize the gene *SiMYB75* in *Arabidopsis thaliana*, we cloned its protein coding region by PCR from sesame root cDNA (F- GCTTTCGCGAGCTCGGTACCATGTCTTGGGGAGTAAT GGG; R-CGACTCTAGAGGATCCTCAATAGAAAGTTGCAGCTA), and inserted the into a pCAMBIA 1301s vector (which is a modified form of the pCAMBIA1301 vector) between KPnI (5’-end) and BamHI (3’-end) sites, driven by the CaMV 35S promoter (**see Supporting Information—****Fig. S1**). The plasmid containing the 35S::*SiMYB75* construct was transformed first into *Agrobacterium tumefaciens* strain LBA4404 and then into *Arabidopsis* ecotype Col-0 cv. Columbia by the floral dipping method (Clough and Bent 1998). Transgenic seeds were screened by sowing on MS medium containing 1 % agar, and 1 % sucrose and 50 μg.ml^-1^ hygromicin. All the putative T1 transgenic plants and wild type (WT) plants were screened by PCR with genomic DNA from leaves as described by Dossa *et al.* (2018) (**see Supporting Information—****Fig. S1**). Furthermore, RT–PCR and β-glucuronidase (GUS) staining were used to confirm the integration of the construct (Wang *et al.* 2009). Three independent T3 transgenic homozygous lines were used for the stress treatments, gene expression assay and phenotypic analyses.

### Sub-cellular localization of SiMYB75 protein

To examine the sub-cellular localization of SiMYB75 protein, the full sequence was loaded in the TargetP 1.1 server with default parameters (Emanuelsson *et al.* 2000) and the WoLF PSORT web site (Horton *et al.* 2007).

In addition, the full-length ORF was PCR amplified and the fragment was ligated into the vector pBWA(V)HS-GLosgfp. Then, the construct pBWA(V)HS-SiMYB75-GLosgfp and the nucleus marker vector (pBWA(V)HS-Nucleus-mKate) were co-transformed into *Arabidopsis* protoplasts under PEG mixture (40 % (w/v) PEG 4000, 2 M mannitol and 0.1 M CaCl_2_). An empty vector, pBWA(V)HS-GFP, was used as a control (Ma *et al.* 2019). After incubation at room temperature for 16–24 h, the expression of plasmid was observed under a laser scanning confocal microscope.

### Evaluation of transgenic lines exposed to ABA, osmotic, salt and drought stresses

First, to analyse the response of the transgenic plants to salt and osmotic stresses, about 30 seeds of WT and three T3 lines (#L1, #L2 and #L3) were surface sterilized and plated on solid Murashige and Skoog (MS) medium containing 0/100 mM mannitol and 0/100 mM NaCl. The seeds were stratified for 2 days in the dark at 4 °C and then transferred to growth chamber at 22 °C under long-day conditions (16 h light/8 h dark). The percentage of germinated seeds with green cotyledons was recorded after 7 days (Manuka *et al.* 2018). Next, 10-days-old over-expressing *SiMYB75* seedlings and WT plants were transferred into solid MS medium supplemented with 0/150 mM mannitol, 0/150 mM NaCl and 0/10 µM ABA. Plates were placed vertically and after 10 days, seedling root length was recorded. In addition, 10-days-old seedlings (transgenic lines and WT) were transferred into pot (two plants per pot) and grown in normal conditions for 15 days. Then, one-third of the pots were subjected to dehydration stress for 17 days. Another one-third of the pots were watered with 200 mM NaCl solution every 2 days for a week. The remaining plants were kept in a normal growth condition throughout the whole experiment. At the end of each stress application, the number of dead/survived plants and rosette biomass dry weight were recorded and pictures were captured to show visible phenotypes. We estimated the relative rosette biomass as the ratio of the records under stress and control conditions. To evaluate the effect of the transgene on the plant growth, daily pictures of the plants were taken starting from 25 days after sowing (beginning of the stress) to 45 days after sowing (after stress treatments). The rosette diameter was estimated; the leaf number and the bolting time were identified. The experiment was repeated once more with four replicates in each experiment for statistical analysis. Leaf samples were collected for physiological, biochemical and gene expression analysis.

### Gene expression analysis in *Arabidopsis*

The qRT–PCR was performed on RNA extracted from leaf samples as described by Dossa *et al.* (2018) using the *Arabidopsis* gene *Actin 2* (*AT3G18780*) as the internal control. Specific primer pairs of *SiMYB75*, four antioxidant genes *AtSOD*, *AtPOD*, *AtAPX*, *AtCAT* and 11 stress marker genes including 11 stress marker genes including *AtRD22*, *AtDREB2C*, *AtABA3*, *AtWRKY28*, *AtNCED3*, *AtEREBP*, *RD29B*, *ATCBF2*, *HSP17.4B*, *ATHSP70–14* and *AtNACO19* were designed (**see Supporting Information—****Table S1**). Samples in the control condition (non-stress) were used as reference and data are presented as relative transcript level based on the 2^-∆∆Ct^ method (Livak and Schmittgen 2001).

### Biochemical analysis

Leaf from salt, drought and control treated plants (five individual plants from each line/treatment) was used for assessing the ABA content. ABA was measured using the high performance liquid chromatography–tandem mass spectrometry (HPLC–MS/MS) method as described by Liu *et al.* (2014). Similarly, for the detection of superoxide (O_2_^-^), leaves were transferred in nitro blue tetrazolium (NBT) (1 mg.ml^-1^) for 5 h. The leaves were boiled in absolute ethanol for 10 min and then pictures were captured for record. Localization of O_2_^-^ was visualized as blue coloration. Leaf tissue was homogenized in 1 mL 80 % methanol, and then centrifuged at 12 000×g for 10 min at room temperature. The ability of the extract to scavenge hydrogen peroxide (H_2_O_2_) was determined according to the method of Ruch *et al.* (1989). Ten microlitres of the extract was mixed with 600 μL of the reaction solution (10 mM of hydrogen peroxide in phosphate buffer, pH = 4). After 10 min of incubation at room temperature, the absorbance was measured at 240 nm. Blank is the reaction solution without hydrogen peroxide. Ascorbic acid was used as a standard. The scavenging effect was expressed in % and calculated:

$$\begin{array}{l} SCAVENGED\;H_{2}O_{2}=100\times \frac{Abs\;\left(control\right)-Abs\;(sample\;or\;standard)}{Abs(sample\;or\;standard)} \end{array}$$

Measurements of protective enzyme activities in leaf samples, including ascorbate peroxidase (APX, EC 1.11.1.11), peroxidase (POD, EC 1.11.1.7), total superoxide dismutase (SOD, EC 1.15.1.1), catalase (CAT, EC 1.11.1.6) as well as the leaf chlorophyll content and the concentration of malondialdehyde (MDA) were determined according to descriptions of Dossa *et al.* (2017c).

### Water loss and stomata closure assays

To examine the possible role of *SiMYB75* in drought stress tolerance, we further conducted water loss experiments using detached leaves (Wang *et al*. 2018a). Four similar shaped and sized leaves were excised from each 4-week-old transgenic and WT lines, and the fresh weight was immediately determined. All leaves were placed on a laboratory bench at room temperature for 210 min. Every 30 min, leaf weight was recorded in the same order that leaves were detached. For the stomata closure assay, the leaves were immersed in a solution containing 10 mM KCl, and 10 mM MES (pH = 6.15) under white light for 3 h. Subsequently, 20 μM ABA was added into the solution for 1 h. In addition, we measured the stomatal movements in leaves detached from drought and salt treated plants. The pictures were taken using a compound microscope with magnification ×40 (OLYMPUS DP72, Japan). We measured more than 100 stomata of each line using the IMAGEJ 1.8.0 software (Broken Symmetry Software). Each experiment was performed in triplicates.

### Cell membrane stability assay

The ion leakage technique was carried out basically as described by Cabello and Chan (2012) on detached leaves at the end of drought and salt treatments. Briefly, detached leaves (~100 mg) were placed in 20 mL of deionized water in two test tubes. One tube was incubated in a water bath at a constant temperature of 40 ºC for 30 min, and its conductivity (C_1_) was measured with a conductivity metre (Orion Star A322). The second tube was placed in a boiling water bath (100 ºC) for 10 min, cooled, and conductivity was recorded (C_2_). Ion leakage was expressed as a percentage using the formula: [1-(C_1_/C_2_)] × 100.

### Statistical analyses

All the data were analysed with the R software (www.r-project.org). One-way analysis of variance was performed by comparing each transgenic line to the wild type plants. This was followed by Tukey HSD test for mean comparison. The error bars were calculated with data from a single experiment.

## Results and Discussion

### SiMYB75 is an R2R3 MYB type gene and is induced by various environmental stresses

**Figure 1.**
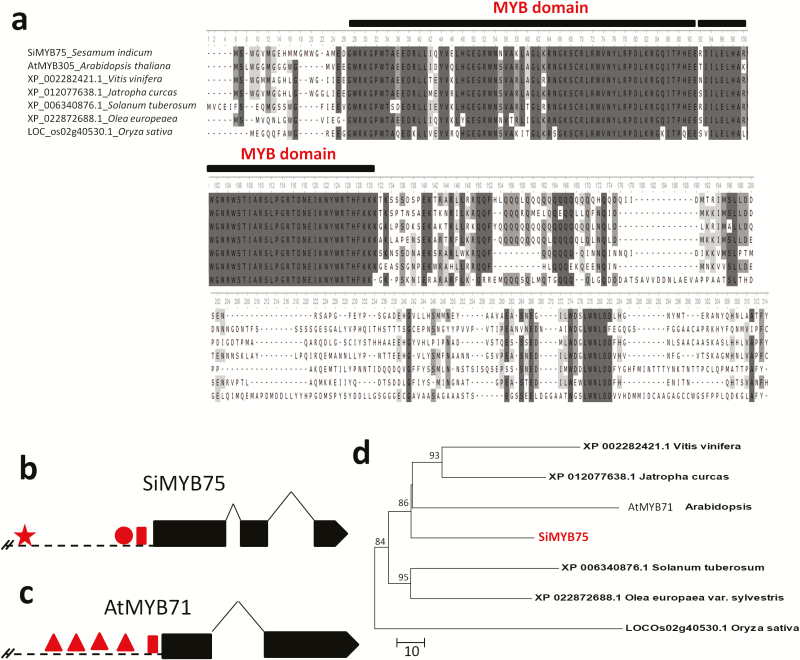
*Sesamum indicum* SiMYB75 amino acid sequence analysis. (**a**) Alignment of deduced protein sequence of SiMYB75 (NCBI protein ID: XP_011073726.1) with those of related proteins in other species including *Arabidopsis thaliana* (National Center for Biotechnology Information (NCBI) protein ID: NP_189074.1), *Solanum tuberosum* (NCBI protein ID: XP_006340876.1), *Japtropha curcas* (NCBI protein ID: XP_012077638.1), *Vitis vinifera* (NCBI protein ID: XP_002282421.1), *Olea europaea* var. sylvestris (NCBI protein ID: XP_022872688.1), *Oryza sativa* (NCBI protein ID: XP_015624171.1). Numbers above indicate the amino acid position and the positions of the two MYB domains are shown; (**b**) Gene structure of *SiMYB75*. Exons and introns are represented by black boxes and lines, respectively. Dashed lines represent 1 Kb promoter region with the position of ABRE motif (red star), the CRT/DRE motif (red circle) and the TATA box (red rectangle); (**c**) Gene structure of *AtMYB71*. Exons and introns are represented by black boxes and lines, respectively. Dashed lines represent 1 Kb promoter region with the position of STRE motifs (red triangle) and the TATA box (red rectangle); (**d**) A phylogenetic tree was constructed in the MEGA7 software with the seven protein sequences using the neighbour-joining method with 1000 bootstrap replicates.

In this study, we retrieved the gene *SIN_1015311* (*SiMYB75*) and found that it contains 2 MYB domains (from 23 to 70 and from 76 to 121 amino acids), indicating an R2R3 MYB type (Fig. 1a). Gene structure analysis showed that *SiMYB75* comprises 3 exons and 2 introns with a total length of 1400 bp (Fig. 1b). The gene is located on the linkage group 3 and encodes an ORF of 256 amino acids with a predicted molecular mass of 29.8 kD and an isoelectric point of 7.92. Prediction of the sub-cellular localization of SiMYB75 using the TargetP 1.1 server and the WoLF PSORT web site indicated that SiMYB75 protein targets the nucleus, similarly as its homolog *AtMYB71* (*At3g24310*) in *Arabidopsis*. Furthermore, using the pBWA(V)HS-SiMYB75-GFP construct transformed into Arabidopsis protoplasts, SiMYB75the GFP signals were co-localized with nuclear marker signals which confirmed that SiMYB75 was localized in the nucleus in accordance with the results predicted by the software (**see Supporting Information—****Fig. S2**). Promoter sequence analysis of *SiMYB75* revealed a TATA box (TATAAA) at the position -142 bp. Furthermore, we identified a CRT/DRE element (ACCGAC) (Dubouzet *et al.* 2003) at the position -243 bp and an ABRE element (ACGTGGC) (Nakashima *et al.* 2006) at the position -914 bp (Fig. 1b). The discovery of a putative functional promoter and presence of stress-related *cis*-acting elements further indicate that *SiMYB75* may serve as an intact gene and function in sesame abiotic stress responses (Ren *et al.* 2016). Similarly, by analysing the promoter region of the homologous gene *AtMYB71* in *Arabidopsis*, we observed four stress response elements (STRE, AGGGG), implying that *AtMYB71* may be also involved in stress responses (Fig. 1c). The construction of the phylogenetic tree based on the alignment of the complete protein sequences of SiMYB75 and its homologues from some related plant species showed that SiMYB75 is more close to its homologues from *Arabidopsis*, jatropha and grape (Fig. 1a and d).

**Figure 2.**
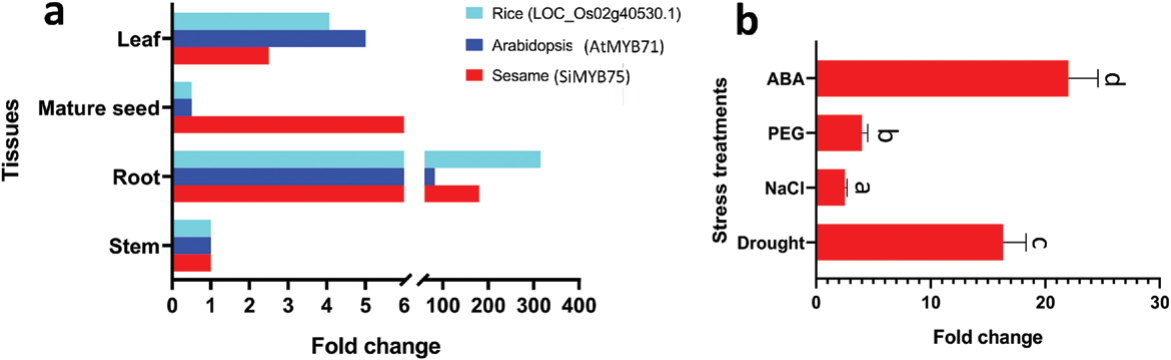
*SiMYB75* expression profiling in tissues and various abiotic stresses. (**a**) Expression in root, seed, leaf and stem of Sesame (www.sesame-bioinfo.org/cgi-bin/Sinbase2.0/search_gene.cgi), *Arabidopsis* (http://bar.utoronto.ca/efp/cgi-bin/efpWeb.cgi?ncbi_accn=822 019&modeInput = Absolute&dataSource=Developmental_Map) and Rice (www.ncbi.nlm.nih.gov/gene/4 330 001). Data expressed as fold change using the stem expression level as the reference; (**b**) Transcript fold change of *SiMYB75* under sodium chloride (NaCl) (200 mM for 48 h), polyethylene glycol (PEG) 6000 (2 % for 5 days), abscisic acid (ABA) (100 µM for 12 h) and drought (water withholding for 7 days) treatments compared with the control treatment in the sesame root. Data shown are average and SD of triplicate quantitative reverse transcriptase polymerase chain reactions. The sesame *Actin 7* gene was used as the internal control. Different letters above bars represent a significant difference between treatments at *P ≤ *0.05.

*SiMYB75* was expressed in various sesame tissues but was more vigorous in the root, implying that *SiMYB75* may play an important role in root growth and development (Fig. 2a). The homologues of *SiMYB75* in *Arabidopsis* (*AtMYB71*) and in rice (*LOC_Os02g40530.1*) were also found strikingly expressed in the root tissue, indicating a conserved spatial expression pattern of this gene in various species (Fig. 2a) (Schmid *et al.* 2005; Wang *et al.* 2015). Moreover, we investigated the expression patterns of *SiMYB75* under different abiotic stresses, including drought, salt, ABA and osmotic stresses, in the sesame root. As expected, we found that *SiMYB75* was strongly induced by all applied stresses, showing that *SiMYB75* may act as a regulator of drought, salt, ABA and osmotic stresses tolerance in sesame. It is worth mentioning that ABA and drought stresses induced significantly higher expression levels of *SiMYB75* as compared to the osmotic and salt treatments (*P < *0.05) (Fig. 2b). Also, osmotic stress higher induced the *SiMYB75* as compared to salt stress. In agreement with our results, *AtMYB71* was found more up-regulated in *Arabidopsis* root under 300 mM mannitol than 150 mM NaCl treatments (Kilian *et al.* 2007). But it was not strongly induced by other abiotic stress treatments such as wounding, UV-B, heat and genotoxic (Kilian *et al.* 2007). Nonetheless, the concentration and duration of osmotic and salt stress treatments were fixed in this study and the possibility that higher expression of *SiMYB75* may be observed at other concentrations or time points cannot be excluded.

Over-expression of SiMYB75 increases tolerance to NaCl, osmotic, drought and confers ABA sensitivity in transgenic Arabidopsis

**Figure 3.**
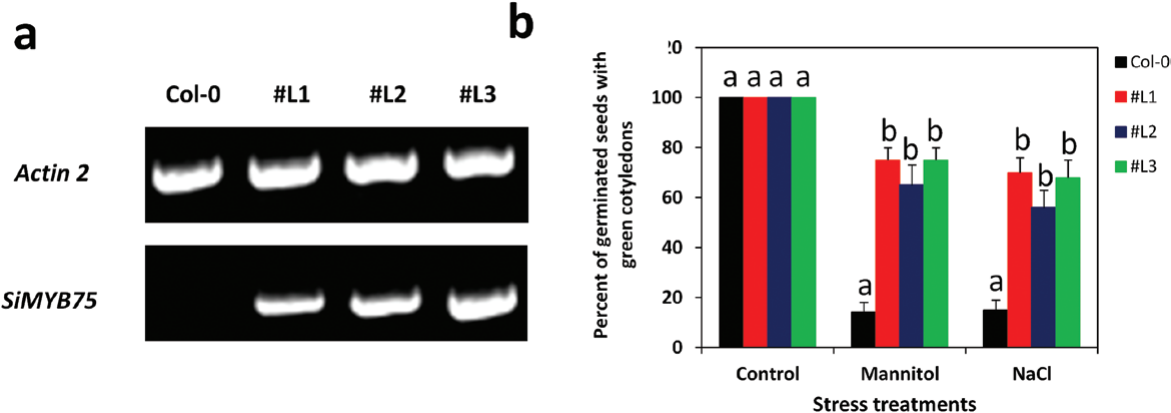
Identification of positive *Arabidopsis* over-expressing lines. (**a**) Reverse transcriptase polymerase chain reaction analysis of *SIMYB75* in the wild type (Col-0) and transgenic lines (#L1, #L2 and #L3).The *Actin 2* gene was used as internal control. (**b**) Evaluation of *Arabidopsis SiMYB75* over-expressing lines (#L1, #L2 and #L3) and wild type plants (Col-0) under mannitol (0/100 mM) and sodium chloride (NaCl) (0/100 mM) treatments at the germination stage. Percentage of germinated seeds with green cotyledons recorded after 7 days. For each experiment, about 30 plants/ lines were used. Data represent means ± SD of five measurements from one experiment and the experiment was repeated once more and similar results were obtained. Different letters above bar mean significant difference between each transgenic line and Col-0 at *P ≤ *0.05.

**Figure 4.**
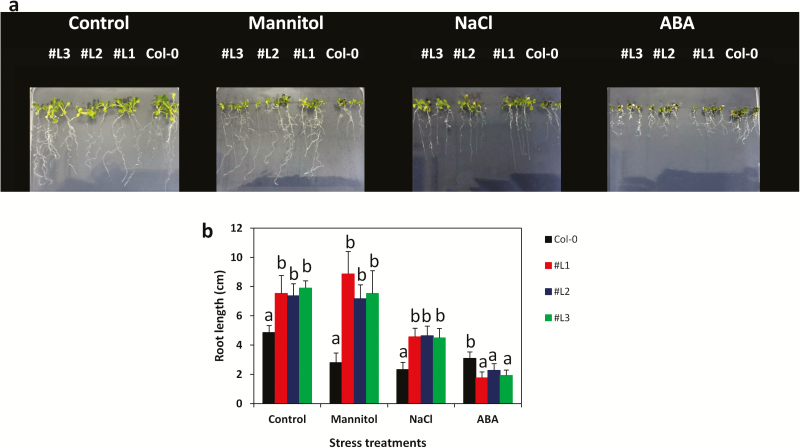
Root growth of *Arabidopsis SiMYB75* over-expressing lines (#L1, #L2 and #L3) and wild type (Col-0) seedlings under various abiotic stress treatments. (**a**) Phenotypes of the transgenic and Col-0 plants under normal Murashige and Skoog (MS) medium and MS medium supplemented with 150 mM mannitol, 150 mM sodium chloride (NaCl) and 10 µM abscisic acid (ABA); (**b**) Root length was recorded after 10 days. For each experiment, 16 seedlings/lines were used. Data represent means ± SD from one experiment and the experiment was repeated once more and similar results were obtained. Different letters above bar mean significant difference between each transgenic line and Col-0 at *P ≤ *0.05.

To study the function of *SiMYB75*, we developed several transgenic *Arabidopsis* lines and selected three independent and homozygous T3 lines (#L1, #L2 and #L3) highly expressing the transgene for functional characterizations. This was confirmed through RT–PCR (Fig. 3a). The three over-expressing *Arabidopsis* lines and the wild-type (WT) plants were subjected to NaCl, mannitol and drought treatments. In the control condition (MS medium), *SiMYB75* over-expressing lines and WT plants germinated well and have green cotyledons (100 %). Under 100 mM mannitol and 100 mM NaCl treatments, the percentage of germinated seeds with green cotyledons was reduced in all lines (Fig. 3b). However, the transgenic plants displayed significantly higher percentages of germinated seeds with green cotyledons than the WT plants (*P ≤ *0.001), indicating that the over-expression of *SiMYB75* imparts osmotic and salt tolerance in *Arabidopsis* at the germination stage. Since *SiMYB75* was found highly expressed in the sesame root, we further analysed the root growth of the transgenic *Arabidopsis* plants under normal and stress conditions (Fig. 4a). In the normal MS medium, the *SiMYB75* over-expressing lines exhibited significantly longer roots than those of WT plants (*P ≤ *0.01) (Fig. 4b). The root length was not affected by mannitol application in the transgenic plants while it was significantly decreased in the WT plants (*P ≤ *0.001) (Fig. 4b). This result suggests that *SiMYB75* promotes root growth maintenance under osmotic stress, which seems to be a tolerance mechanism. The MYB proteins have been described as key components involved in root growth regulation, particularly under abiotic stresses (Baldoni *et al.* 2015). Fang *et al.* (2017) by investigating a novel R2R3 MYB gene *PtrSS1* from poplar found that the transgenic lines increased the lateral root number to resist under salt stress conditions. Similar to our findings, the over-expression of the gene *AtMYB60* increased root growth and conferred tolerance to short-term drought stress in *Arabidopsis* (Oh *et al.* 2011). Moreover, using a phylogenetic approach, Mmadi *et al.* (2017) classified the gene *SiMYB75* in the subgroup C3 together with the gene *AtMYB59*, which was defined as a root development related gene (Mu *et al.* 2009). Under salt treatment, the root growth was reduced in all plants when compared to the growth in the normal MS medium, though, the over-expressing lines showed significantly higher root length than the WT plants (*P ≤ *0.001). This highlights the higher ability of the transgenic lines to cope with the NaCl stress (Fig. 4b). Conversely, we observed an increased ABA sensitivity of the transgenic lines.

**Figure 5.**
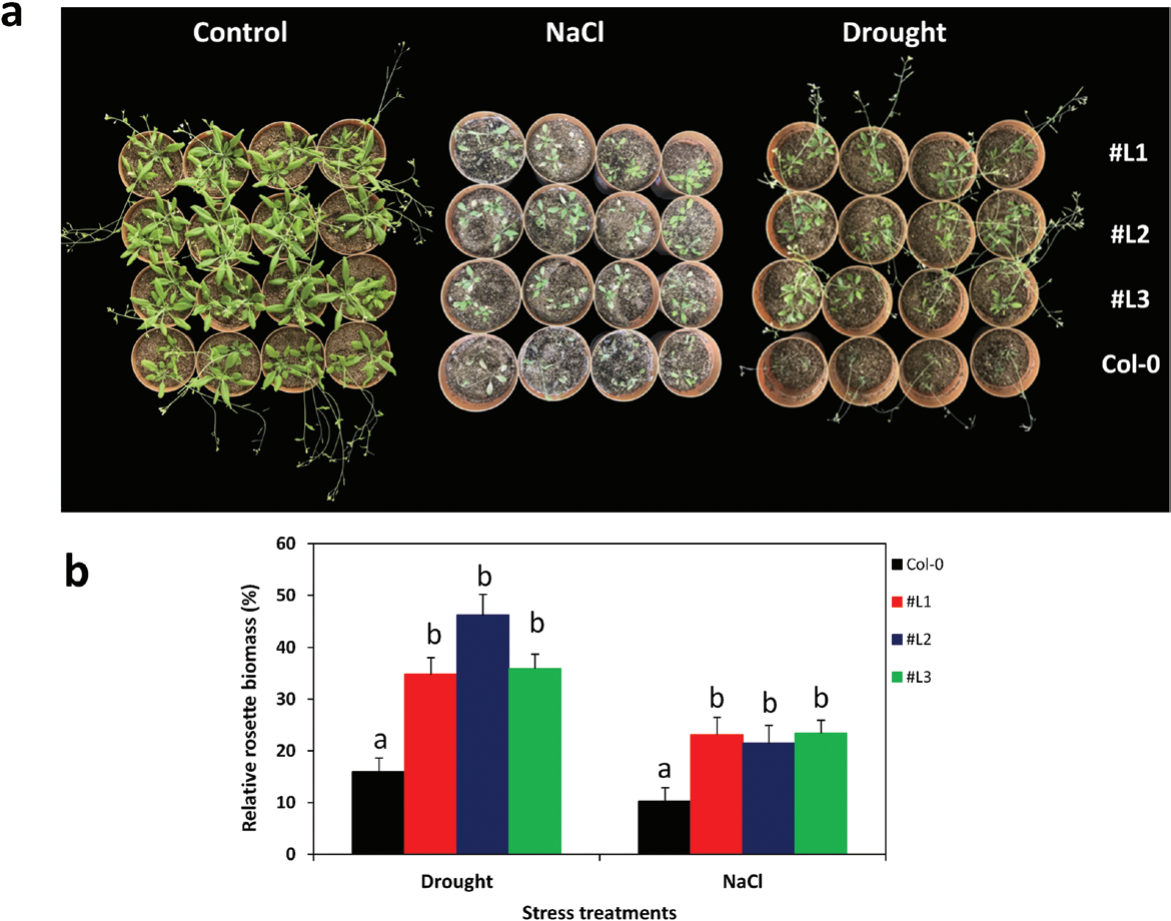
Drought and salt stress tolerance assay at the seedling stage of *Arabidopsis SiMYB75* over-expressing lines (#L1, #L2 and #L3) and wild type plants (Col-0). (**a**) Phenotypes of the transgenic and Col-0 plants in control, sodium chloride (NaCl) (200 mM for 7 days) and drought (17 days water withholding) treatments; (**b**) relative rosette biomass estimated as the ration of the dry weight in the stress and control treatments. For each experiment, 8 plants/lines were used. Data represent means ± SD from one experiment and the experiment was repeated once more and similar results were obtained. Different letters above bar mean significant difference between each transgenic line and Col-0 at *P ≤ *0.05.

Next, we examined the tolerance of the over-expressing *SiMYB75* lines at the seedling stage either under water deprivation (drought) or high NaCl concentration (200 mM) (Fig. 5a). The transgene did not have significant effect on plant size, bolting time and dry matter under normal growth conditions (**see Supporting Information—****Fig. S3**). However under drought and salt stress conditions, the growth of both WT and transgenic plants was inhibited. All plants bolted later, and had lower leaf numbers, rosette diameters and dry weights when compared with the controls. Nonetheless, much less severe effects were observed for the transgenic lines than WT plants (**see Supporting Information—****Fig. S3**). As shown in **Supporting Information—****Table S2**, nearly all the transgenic plants survived 17 days drought stress while most of WT plants died. Similarly, the transgenic lines had higher survival rates than the WT plants under salt stress (**see Supporting Information—****Table S2**). In addition, the transgenic lines were able to maintain a significantly higher biomass production under stress than the WT plants, as shown by the relative rosette biomass under drought (*P ≤ *0.001) and salt (*P ≤ *0.001) (Fig. 5b).

Overall, the *Arabidopsis* transgenic experiment confirmed that *SiMYB75* is a positive modulator of osmotic, drought and salt tolerance. *SiMYB75* seems to be more effective under osmotic/drought stress than salt stress. In fact, plants exposed to salt and drought stresses both suffer from osmotic stress. Additionally, salt stress provokes a strong ion toxicity resulting from a fast ion uptake by the plants (Jakab *et al.* 2005). Therefore, it is probable that *SiMYB75* primarily mediates cellular osmotic adjustment but is not involved in ion toxicity prevention. This finding correlates well with the observed levels of *SiMYB75* induction under drought, osmotic and salt stresses in sesame (Fig. 2b).

### Physiological and biochemical adaptations under drought and NaCl stresses in *SiMYB75* over-expressing *Arabidopsis* lines

**Figure 6.**
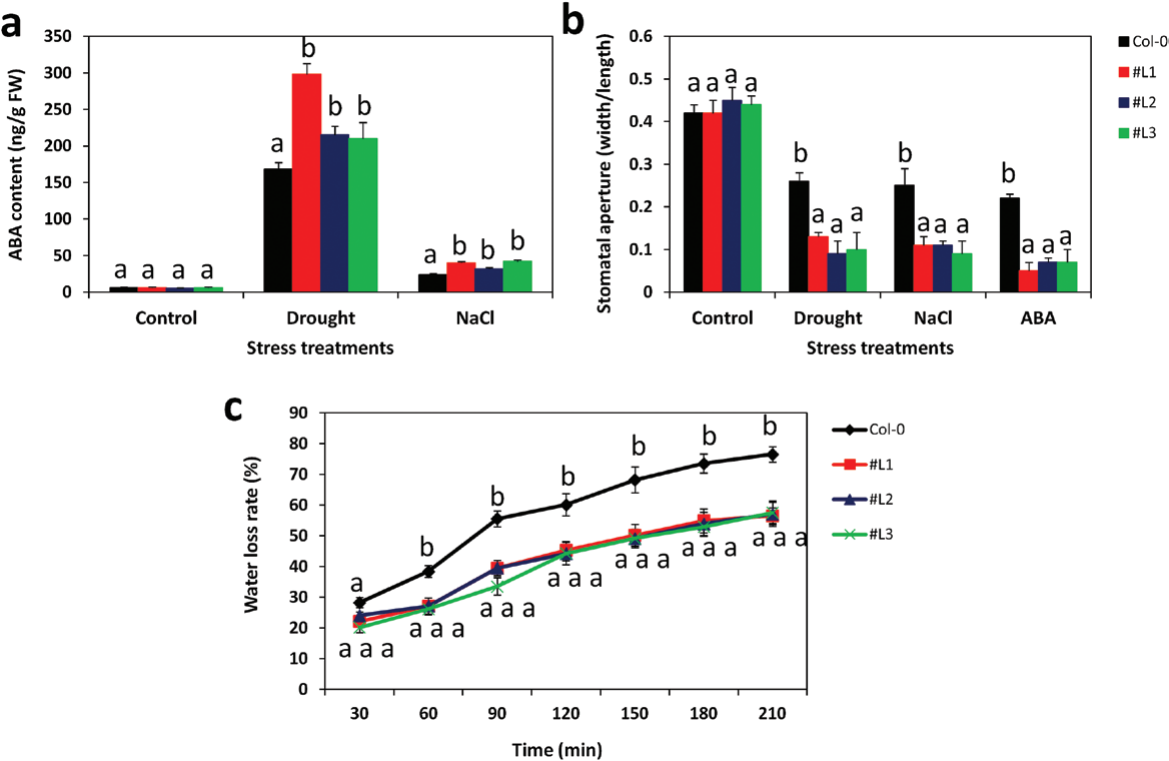
Physiological and biochemical changes in the *SiMYB75* over-expressing lines (#L1, #L2 and #L3) compared to wild type plants (Col-0) under drought and salt treatments. (**a**) abscisic acid (ABA) content in leaves of transgenic and Col-0 plants in control, sodium chloride (NaCl) (200 mM for 7 days) and drought (17 days water withholding) treatments; (**b**) stomata aperture (width/length) assay of transgenic and Col-0 plants in control, drought, salt and 20 µM ABA treatments. Bright-field pictures were taken at ×40 magnification using an OLYMPUS DP72 microscope and over 100 stomata per line/treatment were analysed using the IMAGEJ software; (**c**) Water loss rates in the leaves detached from transgenic and Col-0 plants under normal conditions during a 210-min period; For each experiment, four plants/lines were used. Data represent means ± SD from one experiment and the experiment was repeated once more and similar results were obtained. Different letters above bar mean significant difference between each transgenic line and Col-0 at *P ≤ *0.05.

Under drought and salinity stress conditions, ABA is usually generated in many biological systems (Mittler 2002). We quantified the endogenous ABA content under normal, drought and salt conditions in leaves of the WT and the transgenic plants. As presented in Fig. 6a, *SiMYB75* over-expressing lines accumulated significantly higher content of ABA under stress treatments than the WT plants (*P ≤ *0.001), with a higher accumulation in drought stress than in salt stress treatments. Most stress-responsive MYBs, including *ZmMYB48*, *OsMYB2*, *SbMYB8*, *AtMYB44*, *AtMYB73*, *AtMYB20*, *AtMYB2* and *AtMYB15* (Abe *et al.* 2003; Jung *et al.* 2008; Ding *et al.* 2009; Yang *et al.* 2012; Cui *et al.* 2013; Kim *et al.* 2013; Yuan *et al.* 2015; Wang *et al.* 2017), were reported to regulate plant response to abiotic stress through the ABA-dependent pathways. In this study, we noticed that *SiMYB75* over-expressing lines were sensitive to the exogenous ABA application (Fig. 4a) and they strongly accumulated the endogenous ABA under abiotic stress treatments. Therefore, we deduce that *SiMYB75* also functions through the ABA-mediated signalling pathways.

Increased biosynthesis and accumulation of ABA limit transpirational water loss through adjustment of the stomatal aperture in plants (Farooq *et al.* 2009). Our stomatal closure (width/maximum length) assay revealed that, in contrast to the WT plants, the transgenic lines almost closed the stomata under ABA, drought and salt treatments (Fig. 6b). In addition, the water loss experiment performed on detached leaves showed that during a 210-min period, the WT plants lost rapidly and more intracellular water than the transgenic lines (Fig. 6c). Since, the stomatal closure is an important gateway for plants to decrease water loss, we infer that the low ability of WT plants to regulate the stomata closure under stress has led to an increased water loss and probably to cell damage and death. Our results corroborate well the conclusions from previous studies of Ren *et al.* (2016) and Wang *et al*. (2018) who showed that over-expression of Maize *ZmMYB48* and Wheat *TaCIPK27*, two genes functioning in the ABA-dependent pathways, increased the endogenous ABA levels under drought stress, promoted the stomatal closure and reduced the intracellular water loss in transgenic *Arabidopsis*, resulting in an enhanced drought tolerance.

**Figure 7.**
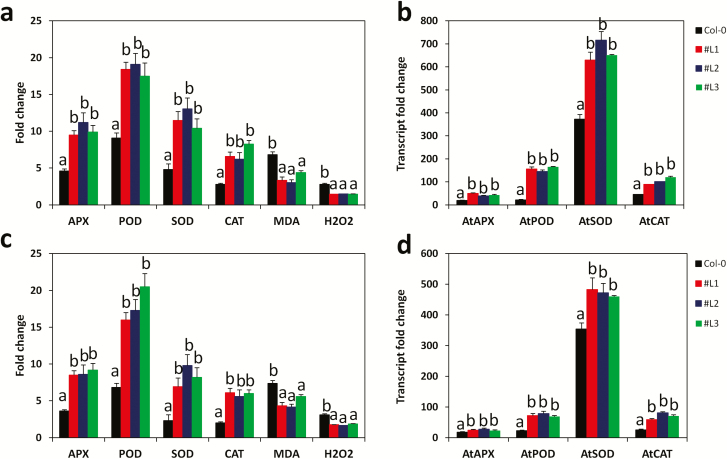
Analysis of the reactive oxygen species (ROS)-scavenging machinery. Enzymes activity (ascorbate peroxidase (APX), superoxide dismutase (SOD), peroxidase (POD), catalase (CAT)) and ROS level indicators (malondialdehyde (MDA), hydrogen peroxide (H_2_O_2_)) in the *SiMYB75* over-expressing lines (#L1, #L2 and #L3) compared to wild-type plants (Col-0) under (**a**) drought and (**c**) salt treatments. Transcript fold change (FC) of four antioxidant genes *AtAPX*, *AtPOD*, *AtCAT* and *AtSOD* under (**b**) drought and (**d**) salt treatments. For each experiment, four plants/lines were used. Data represent means ± SD from one experiment and the experiment was repeated once more and similar results were obtained. Different letters above bar mean significant difference between each transgenic line and Col-0 at *P ≤ *0.05.

**Figure 8.**
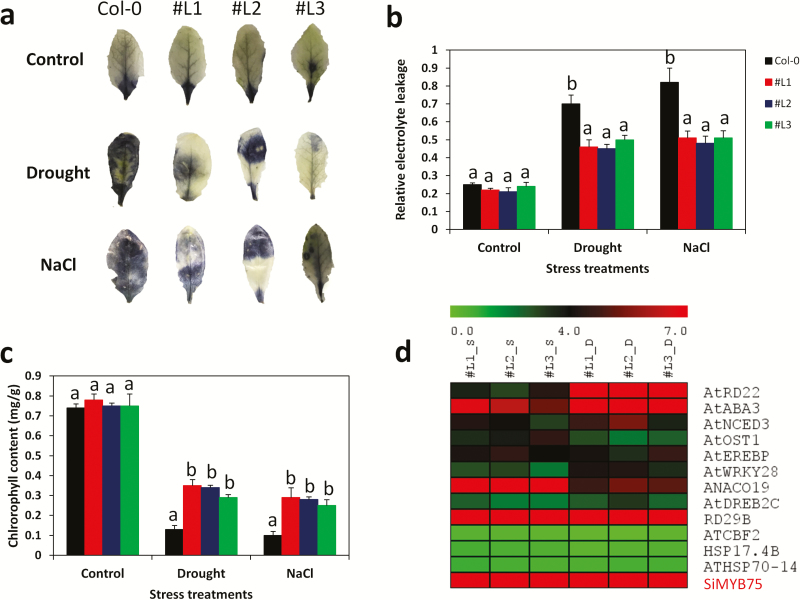
The transgenic lines (#L1, #L2 and #L3) suffered less than wild-type plants (Col-0) from drought and salt. (**a**) Nitro blue tetrazolium staining of leaves detached from plants under control, drought and sodium chloride (NaCl) treatments. (**b**) Electrolyte leakage. (**c**) Measurement of chlorophyll content. For each experiment, four plants/lines were used. Data represent means ± SD from one experiment and the experiment was repeated once more and similar results were obtained. Different letters above bar mean significant difference between each transgenic line and Col-0 at *P ≤ *0.05. (**d**) Heatmap displaying fold changes (FC) of abiotic stress marker genes and abscisic acid (ABA) signalling and biosynthesis genes in the transgenic lines compared to Col-0 plants (FC transgenic line/ FC wild-type plants) under drought and NaCl treatments based on quantitative reverse transcriptase polymerase chain reaction. The *Actin 2* gene was used as internal control. Data shown are average of triplicate reactions. S and D represent salt and drought treatments, respectively.

In plants, drought and salinity stresses cause oxidative damage via the production of reactive oxygen species (ROS), such as H_2_O_2_ and superoxide (Mittler 2002; Xiong and Zhu 2002). High ROS-scavenging activities decrease the over-accumulation of ROS in plants, thereby inhibiting the onset of programmed cell death (Mittler 2002; Farooq *et al.* 2009). To investigate the ROS-scavenging activities in the transgenic lines and WT plants, the levels of the enzymes SOD, POD, CAT and APX and their corresponding transcripts *AtSOD*, *AtPOD*, *AtCAT* and *AtAPX* (Gao *et al.* 2012) were evaluated under drought (Fig. 7a and b) and salt (Fig. 7c and d). For the entire assayed enzymes, we observed an increase in their activities under stress compared to the control conditions. Similar observations were noted for their corresponding transcript levels. However, these inductions were significantly higher in the transgenic lines than in WT plants. These results denote that the over-expressing lines have an elevated ROS scavenging ability as a potent mechanism underlying their strong tolerances to drought and salt stresses. The molecule malondialdehyde (MDA) has been associated with lipid peroxidation via an increased generation of ROS, and thus its quantification has been suggested as a general indicator for stress tolerance (Dossa *et al.* 2017c). We, therefore, analysed the MDA and H_2_O_2_ contents in leaf tissues under stress conditions in relation to the control treatment (Fig. 7a and c). Our results showed that the stress-induced accumulations of MDA and H_2_O_2_ in cells of WT plants were significantly higher than in cells of the transgenic lines (*P ≤ *0.001). This implies that WT plants suffered more from stresses than the transgenic lines. Fig. 8a showed that there was no difference between the WT plants and the transgenic lines regarding the superoxide production under control condition. However, under drought and salt stress treatments, WT plants produced more superoxide than transgenic lines further providing a solid support to our previous results. Next, we examined the relative electrolyte leakage and the chlorophyll content in leaves of stressed and control plants (Fig. 8b and c). Both drought and salt increase the cell ion leakage while decrease the chlorophyll contents in all plants. By comparing the WT plants and the transgenic lines, we found that the transgenic lines suffered lower cell membrane damage than the WT counterparts. On the other hand, the chlorophyll content was significantly higher in the transgenic lines compared to the WT plants (*P ≤ *0.001)

In summary, our physiological and biochemical investigations established that over-expression of *SiMYB75* increases ABA content under stress conditions, which leads to stomatal closure and reduced water loss in the leaves. In addition, *SiMYB75* acts through the ROS signalling pathway by increasing the activity of antioxidant enzymes and probably other non-enzymatic components to reduce ROS production and alleviate cell damage and apoptosis.

### Over-expression of *SiMYB75* increases the expression of abiotic stress-responsive genes in the transgenic plants

Under adverse conditions, transcription factors can induce or repress the activity of the RNA polymerase, thus regulating target gene expression in plants (Saibo *et al.* 2009). Since, *SiMYB75* over-expressing lines displayed improved tolerance to drought, salt and osmotic stresses through ABA-mediated pathways in transgenic *Arabidopsis* plants, we hypothesized that *SiMYB75* modulates the expression levels of downstream abiotic-stress-marker genes in *Arabidopsis*. To test this hypothesis, we performed qRT–PCR analysis of stress-marker genes related to salinity, cold, heat, drought and genes related to ABA biosynthesis, e.g. *AtRD22*, *AtABA3*, *AtNCED3*, *AtOST1*, *AtEREBP*, *AtWRKY28*, *ANAC019*, *RD29B*, *ATCBF2*, *HSP17.4B*, *ATHSP70–14* and *AtDREB2C* (Swindell *et al.* 2007; Manuka *et al.* 2018; Park *et al.* 2018), under control, drought and salt treatments. The results revealed that drought and salt treatments increased the expression levels of all tested stress marker genes in WT and transgenic lines. However, by comparing the fold changes (FC) of the expression levels of those genes in the transgenic lines with the WT plants (FC transgenic lines/FC WT plants), we observed that all the tested stress marker genes were up-regulated in the transgenic lines under both stress treatments except *ATCBF2, HSP17.4B* and *ATHSP70–14* (Fig. 8d).

Previous studies demonstrated that the expression levels of the genes *AtNCED3* and *AtABA3* involved in ABA synthesis increased under abiotic stresses such as drought and salinity (Yamaguchi-Shinozaki *et al.* 2006; Nakashima *et al.* 2009; Xie *et al.* 2014). Our results are in accordance with these reports. It implies that the elevated concentration of ABA in *SiMYB75* over-expressing lines (Fig. 6a) is favoured by the increased expression levels of ABA synthesis genes. *RD29B* which is the main ABA-dependent pathway marker gene (Msanne *et al.* 2011) was found strongly up-regulated in the transgenic lines as compared to the wild-type plants, further supporting the premise that *SiMYB75* over expression acts in the ABA-dependent pathway. According to Yunta *et al.* (2011), an increase in ABA content stimulates within minutes the regulators such as the *OPEN STOMATA 1* (*OST1*) gene, which modulates the stomatal closure. Therefore, we interpreted that the over-expression of *SiMYB75* further triggered the expression level of *AtOST1* in the transgenic lines under stress treatments resulting in a rapid stomatal closure (Fig. 6b). The *RESPONSIVE TO DEHYDRATATION* 22 (*RD22*) gene is used as a reliable ABA early response marker in plants (Yamaguchi-Shinozaki *et al.* 1993; Matus *et al.* 2014). The *Arabidopsis* gene *AtRD22* is up-regulated by moisture stress, salinity and exogenously applied ABA (Yamaguchi-Shinozaki *et al.* 1993). Wang *et al.* (2012) also demonstrated that the soybean *GmRD22* gene could directly improve salt stress tolerance when over-expressed in both *Arabidopsis* and rice. Similarly, the strong up-regulation of several stress-induced transcription factors such as *AtEREBP*, *AtWRKY28*, *ANAC019* and *AtDREB2C* were shown to be a tolerance mechanism upon exposure to abiotic stresses in *Arabidopsis* (Tran *et al.* 2004; Babitha *et al.* 2013; Liu *et al.* 2013; Manuka *et al.* 2018; Wang *et al*. 2018). Interestingly, the three genes (*ATCBF2*, *HSP17.4B* and *ATHSP70–14*) that did not exhibit significant difference between transgenic lines and WT plants, have been shown to regulate different abiotic stresses such as heat and cold and are not induced by ABA (Swindell *et al.* 2007; Park *et al.* 2018). In that sense, our results validate the formulated hypothesis referring to the participation of *SiMYB75* in the ABA-dependent pathways and indicate that the enhanced ability to cope with drought, salt and osmotic stresses of the *SiMYB75*-over-expressing lines is due in part to the increased expression of these abiotic stress-marker genes.

## Conclusions

In this report, we cloned and characterized a novel R2R3-MYB gene *SiMYB75* from sesame. We demonstrated that *SiMYB75* works through the ABA-mediated pathways and positively modulates drought, salt and osmotic stresses responses. These results increase our understanding of the roles of sesame MYB transcription factors in response to abiotic stresses. *SiMYB75* is, therefore, a promising candidate gene for sesame crop improvement. However, the difficulties related to the sesame genetic transformation hinder the development of genetically modified cultivars with improved performances in field. Nonetheless, to harness the potential of *SIMYB75*, we will study the natural variation in its promoter and genic regions in association with drought and salt stress responses in a large panel of sesame (Dossa *et al.* 2019a). This will help identify superior haplotypes and develop specific molecular markers for breeding applications.

## Supporting Information

The following additional information is available in the online version of this article—

**Table S1.** List of the primers used for qRT-PCR experiments in sesame and *Arabidopsis*

**Table S2.** Number of survived and dead plants aftr 17 days drought and 7 days under 200 mM NaCl treatments. For each experiment, 8 plants/ lines were used.

**Fig. S1.** Map of the vector used for the transformation of *Arabidopsis* and PCR results of the T1 transgenic plants. M represents 2 Kb DNA Ladder.

**Fig. S2.** Subcellular localization of the SiMYB75 protein in *Arabidopsis* protoplasts. A, fluorescence signal in target protein co-localizations with a Nucleus marker; B, fluorescence signal of the GFP empty vector.

**Fig. S3.** Growth parameters of transgenic *SiMYB75* lines and its Wild Type (WT) under control, drought and salinity conditions, including leaf number, rosette diameter, day to bolting, rosette dry weight at 25 days after sowing (DAS) and at 45 DAS. Data represent means ± SD of five measurements from one experiment and the experiment was repeated once more and similar results were obtained. Different letters above bar mean significant difference between each transgenic line and Col-0 at *P ≤ 0*.05.

plz081_suppl_Supplementary_Table_S1

plz081_suppl_Supplementary_Table_S2

plz081_suppl_Supplementary_Figure_S1

## Notes

Dossa K, Mmadi MA, Zhou R, Liu A, Yang Y, Diouf D, You J and Zhang X. 2019. Ectopic expression of the sesame MYB transcription factor *SiMYB305* promotes root growth and modulates ABA-mediated tolerance to drought and salt stresses in *Arabidopsis.* AoB PLANTS **11**: plz081; doi: 10.1093/aobpla/plz081

## Data

All data generated or analysed during this study are included in this published article and its supplementary information files.
