# Supplementary material for: Ectopic expression of the sesame MYB transcription factor SiMYB305 promotes root growth and modulates ABA-mediated tolerance to drought and salt stresses in Arabidopsis
Source: AoB Plants. 2019 Dec 24;12(1):plz081. doi: 10.1093/aobpla/plz081 (PMC7019004; doi:10.1093/aobpla/plz081)

Figure S1 Map of the vector used for the transformation of *Arabidopsis* and PCR results of the T1 transgenic plants. M represents 2 Kb DNA Ladder.

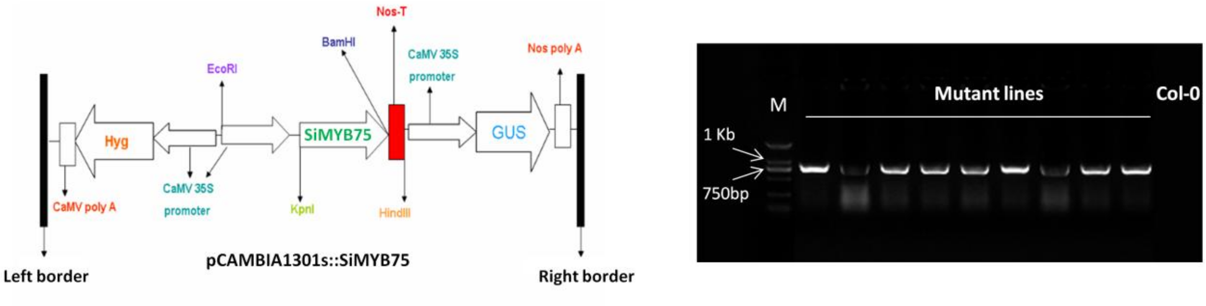

Figure S2 protein co-localizations with a Nucleus marker; B, fluorescence signal of the GFP empty vector.

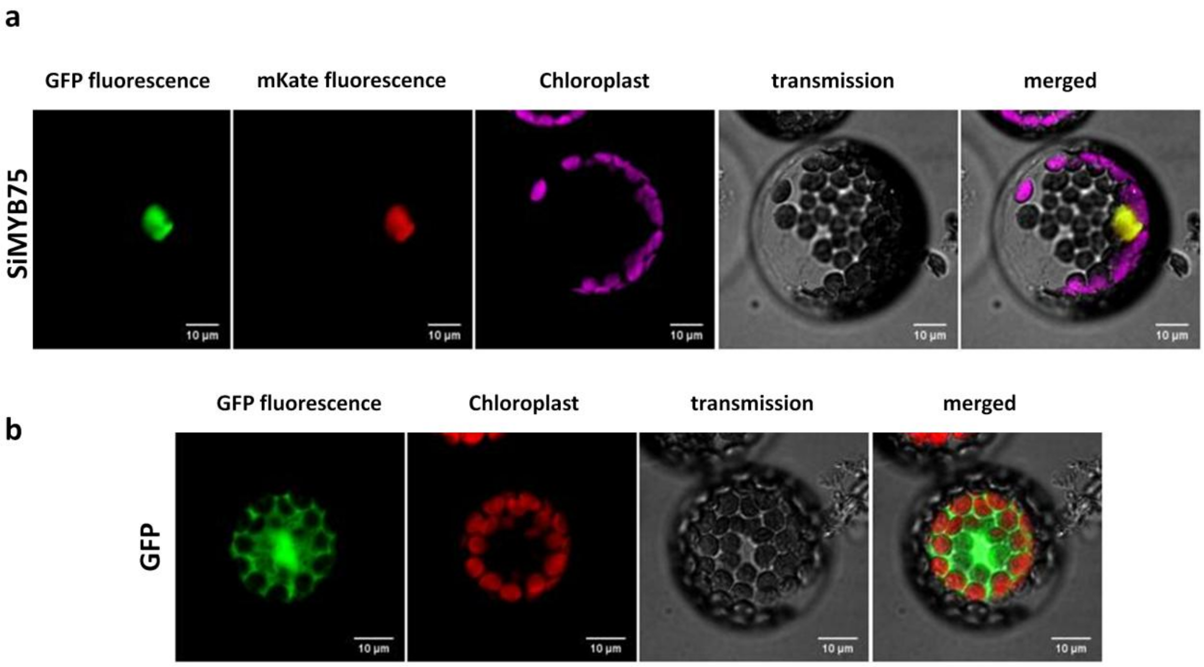

Figure S3 Growth parameters of transgenic *SiMYB75* lines and its Wild Type (WT) under control, drought and salinity conditions, including leaf number, rosette diameter, day to bolting, rosette dry weight at 25 days after sowing (DAS) and at 45 DAS. Data represent means ±SD of five measurements from one experiment and the experiment was repeated once more and similar results were obtained. Different letters above bar mean significant difference between each transgenic line and Col-0 at $P \leq 0.05$.

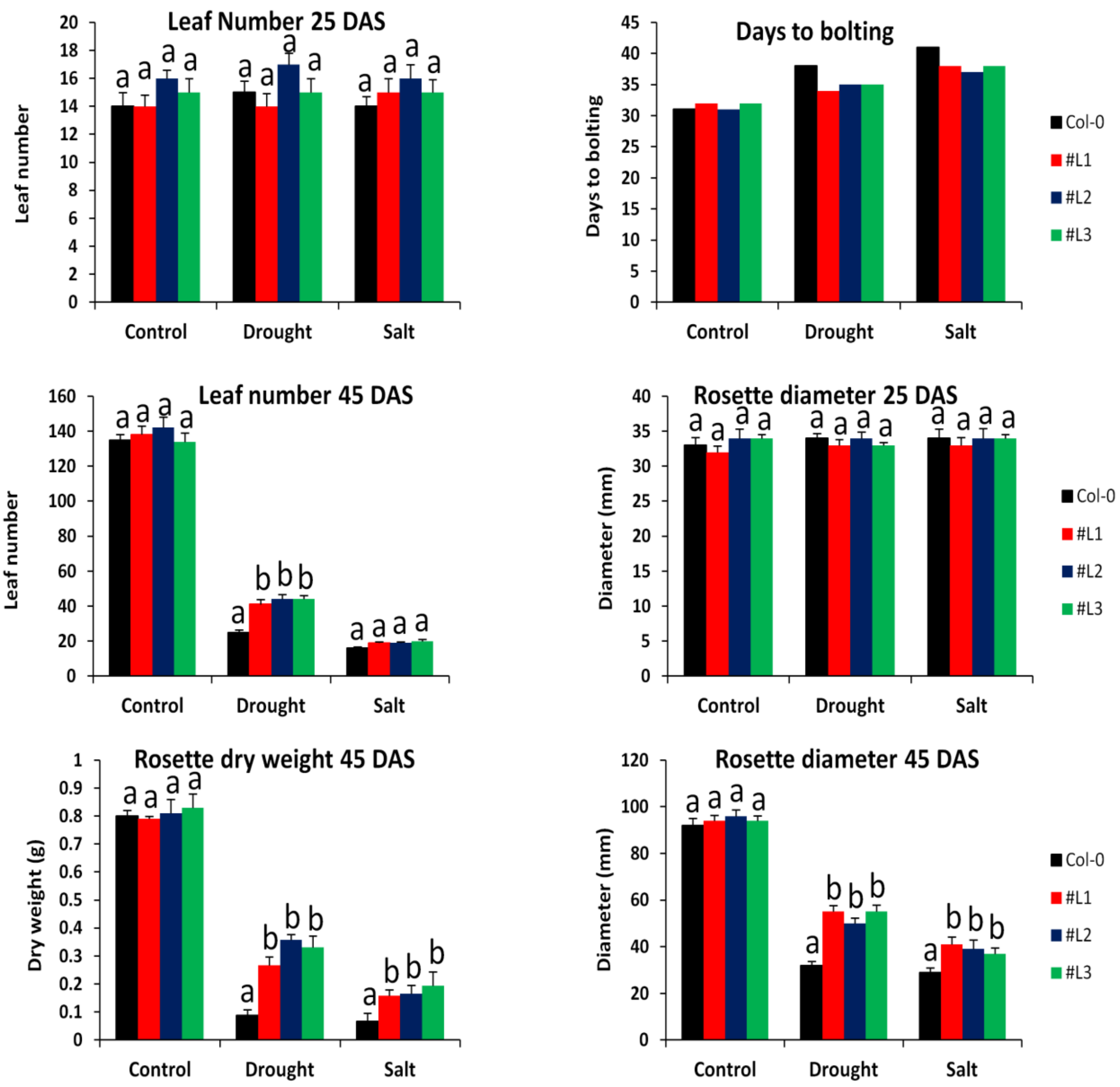

Supplement: plz081_suppl_Supplementary_Figure_S1 [file plz081_suppl_supplementary_figure_s1.pdf]
